# The legacy of Eastern Mediterranean mountain uplifts: rapid disparity of phylogenetic niche conservatism and divergence in mountain vipers

**DOI:** 10.1186/s12862-021-01863-0

**Published:** 2021-06-22

**Authors:** Mohsen Ahmadi, Mahmoud-Reza Hemami, Mohammad Kaboli, Masoud Nazarizadeh, Mansoureh Malekian, Roozbeh Behrooz, Philippe Geniez, John Alroy, Niklaus E. Zimmermann

**Affiliations:** 1 Department of Natural Resources, Isfahan University of Technology, 84156-83111 Isfahan, Iran; 2 Department of Environmental Science, Faculty of Natural Resources, University of Tehran, Karaj, Iran; 3 Department of Parasitology, Faculty of Science, University of South Bohemia, České Budějovice, Czech Republic; 4 Institute of Parasitology, Biology Centre CAS, v.v.i., České Budějovice, Czech Republic; 5 CEFE, PSL-EPHE (Biogéographie et Ecologie des Vertébrés), CNRS, University of Montpellier, Montpellier, France; 6 Department of Biological Sciences, Faculty of Science and Engineering, Macquarie University, Macquarie Park, NSW 2109 Australia; 7 Swiss Federal Research Institute WSL, CH-8903, Birmensdorf, Switzerland

**Keywords:** Allopatric speciation, Biogeography, Divergence dating, Diversification, Mountain orogeny, Niche evolution, Niche modelling, Near East, Middle East, *Montivipera*

## Abstract

**Background:**

The orogeny of the eastern Mediterranean region has substantially affected ecological speciation patterns, particularly of mountain-dwelling species. Mountain vipers of the genus *Montivipera* are among the paramount examples of Mediterranean neo-endemism, with restricted ranges in the mountains of Anatolia, the Levant, Caucasus, Alborz, and Zagros. Here we explore the phylogenetic and ecological diversification of *Montivipera* to reconstruct its ecological niche evolution and biogeographic history. Using 177 sequences of three mitochondrial genes, a dated molecular phylogeny of mountain vipers was reconstructed. Based on 320 occurrence points within the entire range of the genus and six climatic variables, ecological niches were modelled and used to infer ancestral niche occupancy. In addition, the biogeographic history and ancestral states of the species were reconstructed across climate gradients.

**Results:**

Dated phylogenetic reconstruction revealed that the ancestor of mountain vipers split into two major clades at around 12.18 Mya followed by multiple vicariance events due to rapid orogeny. *Montivipera* colonised coastal regions from a mountain-dwelling ancestor. We detected a highly complex ecological niche evolution of mountain vipers to temperature seasonality, a variable that also showed a strong phylogenetic signal and high contribution in niche occupation.

**Conclusion:**

Raising mountain belts in the Eastern Mediterranean region and subsequent remarkable changes in temperature seasonality have led to the formation of important centres of diversification and endemism in this biodiversity hotspot. High rates of niche conservatism, low genetic diversity, and segregation of ranges into the endemic distribution negatively influenced the adaptive capacity of mountain vipers. We suggest that these species should be considered as evolutionary significant units and priority species for conservation in Mediterranean mountain ecosystems.

**Supplementary Information:**

The online version contains supplementary material available at 10.1186/s12862-021-01863-0.

## Background

The recent decline in biodiversity necessitates a deeper understanding of current species distribution patterns and their evolution through time. This knowledge is particularly important in ecology, evolution, and conservation planning [1, 2]. In this regard, limited resources for nature conservation need to be concentrated on species having unique evolutionary histories [3, 4]. One of the main prerequisites for maintaining a species in the face of global change, and specifically climate change, is having sufficient genetic diversity to support environmental tolerances and occupy different ranges of ecological conditions (i.e., niche evolution), which is referred to as evolutionary flexibility or adaptive capacity [5, 6]. Species that have slowly evolved their ecological niches in the past, i.e., represent high degrees of niche conservatism [7], are more sensitive to demographic changes and population declines [6]. Significant efforts have been made recently to identify and model characteristics that allow species to adapt to environmental changes [e.g. 8, 9]. However, for many species, detailed demographic information and local adaptation rates are unavailable. A practical tool in this regard is to combine comparative phylogenetic methods and ecological niche modelling (ENM) to shed light on differential rates of niche evolution among sister taxa [6, 10, 11].

Ecological divergence has been emphasised as one of the main drivers of speciation [12, 13]. During ecological divergence, natural populations are influenced by a variety of biotic and abiotic factors, such as inter- and intra-species interactions (e.g., predation, competition) and fluctuations in climate and food resources [14, 15]. This may result in separate evolutionary responses and the formation of distinct climatic tolerances (i.e., niche divergence) [12, 16, 17]. In contrast, preservation of similar ecological conditions over time, in line with allopatric speciation, limits adaptation to local conditions and confines genetic differentiation between vicariant populations (i. e., niche conservatism) [18–20].

Mountains are one of the main cradles of diversification, as half of the world’s biodiversity hotspots are located in mountainous landscapes [21, 22]. A tremendous variety of microclimates and diverse local conditions in mountain ranges facilitates local adaptation and the formation of distinct genetic structures during ecological speciation [23, 24]. However, fragmentation and isolation in populations of mountain-dwelling species lead to limited gene flow, resulting in allopatric and peripatric speciation during orogeny [25, 26] that do not require niche adaptation as a motivator. Such processes play an important role, particularly in mountains that undergo rapid orogeny, where newly emerging extreme environments provide room for generating regional diversification pulses [22, 24].

**Fig. 1:**
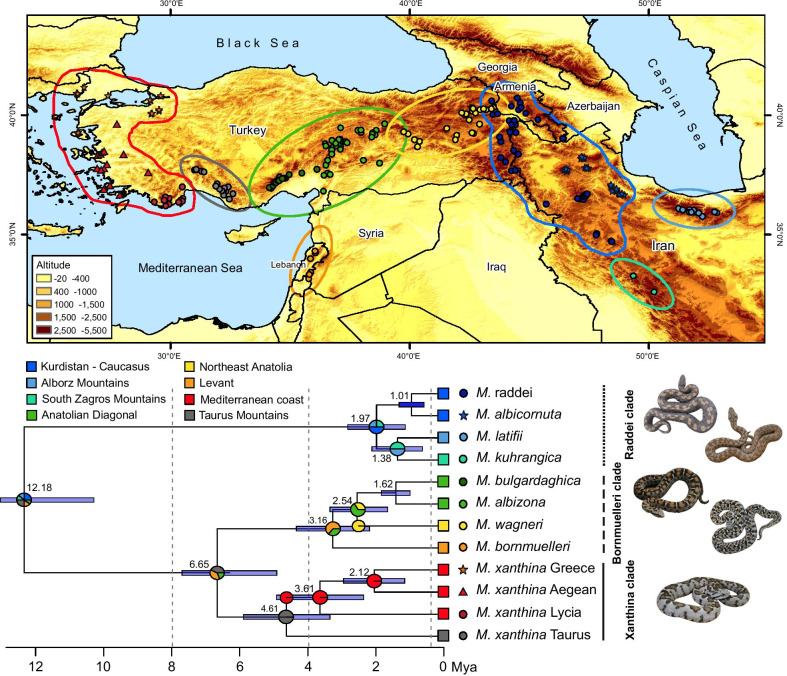
Geographic distribution (top), dated maximum clade credibility tree (bottom, reconstructed in BEAST based on multi-locus mitochondrial data), and ancestral range estimates (inferred with BIOGEOBEARS using a DIVA-like model) of the genus *Montivipera*. Numbers at each node indicate mean age estimates, and blue bars the 95% highest posterior density (HPD) confidence intervals. Boxes at tips show geographic areas including each species, colour-coded for the eight areas illustrated as polygons on the map. Pie charts indicate the estimated probability of each area. Dots, triangles, and stars next to the phylogenetic tree is the species-specific indication of the occurrence points and coloured polygons on the map are the approximate range of the biogeographic areas

**Fig. 2:**
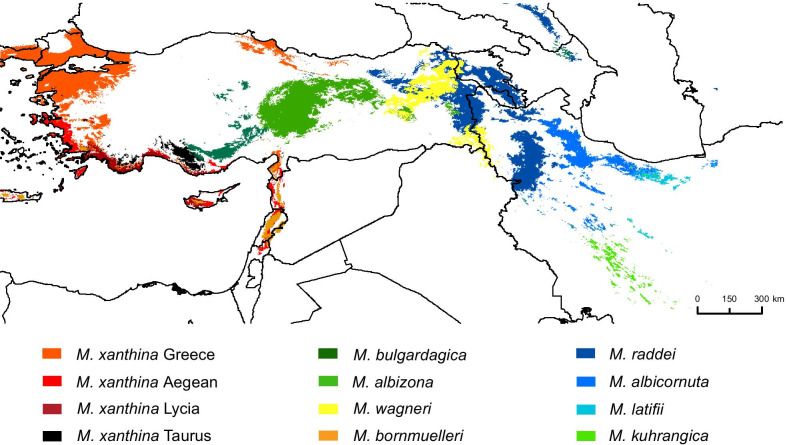
Geographic distribution of suitable habitats of mountain vipers predicted by the distribution models and used for the ancestral niche occupancy reconstruction. Potential distributions were modelled using six climatic variables

Our research focuses on one such mountain system, the Iran-Anatoly-Caucasus mountain chains, a long and newly developed rugged landscape that stretches from the Taurus Mountains in the south of Turkey, elongates toward the Anatolian Diagonal to the Caucasus and ends at the Alborz and Zagros Mountains in the north and southwest of Iran (Fig. 1). In general, the interaction of tectonic, topographic, and climatic physiognomies has shaped a heterogeneous assemblage of biodiversity in this region [26, 27]. This area embraces two biodiversity hotspots, the Caucasus and Irano-Anatoly, characterised by high degrees of endemism [28]. Moreover, severe ecological conditions have resulted in a repetitive pattern of expansion, contraction, and isolation in the Irano-Anatolian and Caucasus regions, and accordingly, the emergence of a substantial degree of recent speciation events, i.e. neo-endemism. Consequently, this region includes one of the world’s richest herpetofauna and preserves the highest diversity of true vipers within the Palearctic biome [29]. One of the striking models of neo-endemism in the Irano-Anatoly and Caucasus regions are mountain vipers of the genus *Montivipera*, which have experienced considerable speciation in mountainous landscapes [26, 30]. Their global distribution includes isolated populations of two species complexes, the Xanthina and the Raddei complex. The Xanthina complex includes the species *M. xanthina*, *M. wagneri,* and *M. bornmuelleri,* and a clade of two closely-related sister species *M. albizona* and *M. bulgardaghica*, although *M. albizona* has also been considered as a subspecies of *M. bulgardaghica* based on the low genetic divergence between them [26]. The Raddei complex contains the species *M. raddei*, *M. latifii*, *M. albicornuta* and, *M. kuhrangica*. While in the Xanthina complex *M. xanthina* ranges from the semi-elevated uplands of southern Turkey to low-elevated plains and islands of the Mediterranean Sea, other species of both complexes disperse in mountain-top isolated populations (Figs. 1, 2). Despite a wealth of studies on the morphology and phylogeny of vipers in general, and mountain vipers in particular, their biogeographic history remains to be more carefully analysed [26, 30, 31].

Given the complex dynamics and idiosyncratic paleontological history of the mountain belts of the Irano-Anatoly and Caucasus regions and the taxonomic controversy over mountain vipers, the main question to be answered is how past changes in environmental conditions have driven the divergence among these species. Specifically, we aimed to unveil that whether the formation of the eastern Mediterranean mountain belt and the consequent climate changes is associated with cladogenic niche divergence. We also intended to explore if and where this orogeny has forced closely related taxa to preserve ecological adaptations after vicariant speciation, i.e., niche conservatism. We further aimed to examine patterns of phylogenetic niche conservatism in range-restricted *Montivipera* species allowing us to identify those species with the highest vulnerability to environmental change. To test these hypotheses, we reconstructed a dated phylogenetic tree and combined evolutionary analyses with ecological niche modelling to explore patterns of niche evolution among sister taxa of the mountain vipers.

## Results

### Phylogenetic relationships and divergence dating

The dated phylogenetic tree of mountain vipers (Fig. 1) allowed the identification of two *Montivipera* groups, i.e., the Xanthina and Raddei complexes, estimated to have diverged around 12.18 Mya (95% HPD 9.9–14.46). The Raddei complex consists of four species, *M. raddei*, *M. albicornuta*, *M. latifii*, and *M. kuhrangica*, showing a strongly supported monophyly that has split around 1.97 Mya (HDP = 0.79–3.15). The species inside this complex have diverged between 1.38 Mya (95% HDP = 0.64–2.12) and 1.01 Mya (95% HDP = 0.62–1.39). A strong cladogenic divergence is visible in the Xanthina complex, with the complex having split into the Xanthina and Bornmuelleri clades around 6.65 Mya (95% HDP = 5–8.3). A comparably early split in the Xanthina clade was the divergence of the coastal groups (*M. xanthina* Lycia, *M. xanthina* Aegean, and *M. xanthina* Greece) from their mountain-dwelling sister group (*M. xanthina* Taurus) at 4.49 Mya (95% HDP = 3.47–5.51). In the Bornmuelleri clade, consisting of *M. bornmuelleri*, *M. wagneri*, *M. albizona* and *M. bulgardaghica*, the divergence from the most recent common ancestor (MRCA) was estimated at 3.16 Mya (95% HDP = 2.18–4.14), 2.54 Mya (95% HDP = 1.73–3.35), and 1.62 Mya (95% HDP 1.42–1.82), subsequently. The complete unpruned phylogenetic tree of the species is shown in the Additional file 1: Fig. S1.

### ENM and history of niche evolution

The distribution models based on six bioclimatic variables resulted in spatially well-defined potential ranges for each of the mountain viper species (Fig. 2). AUC and TSS values obtained for the distribution models among the eight studied species ranged from 0.88 and 0.89 in *M. xanthina* to 0.95 and 0.94 in *M. bornmuelleri*, respectively (Additional file 1: Table S2). The contribution of climatic variables to explain their distribution varied among species. Yet, temperature seasonality, temperature of the warmest month, precipitation seasonality, and precipitation of the driest month revealed the highest levels of importance for almost all species (Additional file 1: Table S2). As a general rule, all *Montivipera* species prefer areas with relatively cold climate conditions and with relatively high temperature seasonality, except for *M. bornmuelleri* and coastal lineages of *M. xanthina*, which show strong preferences for comparably warmer climates having low temperature seasonality and high precipitation seasonality.

**Fig. 3:**
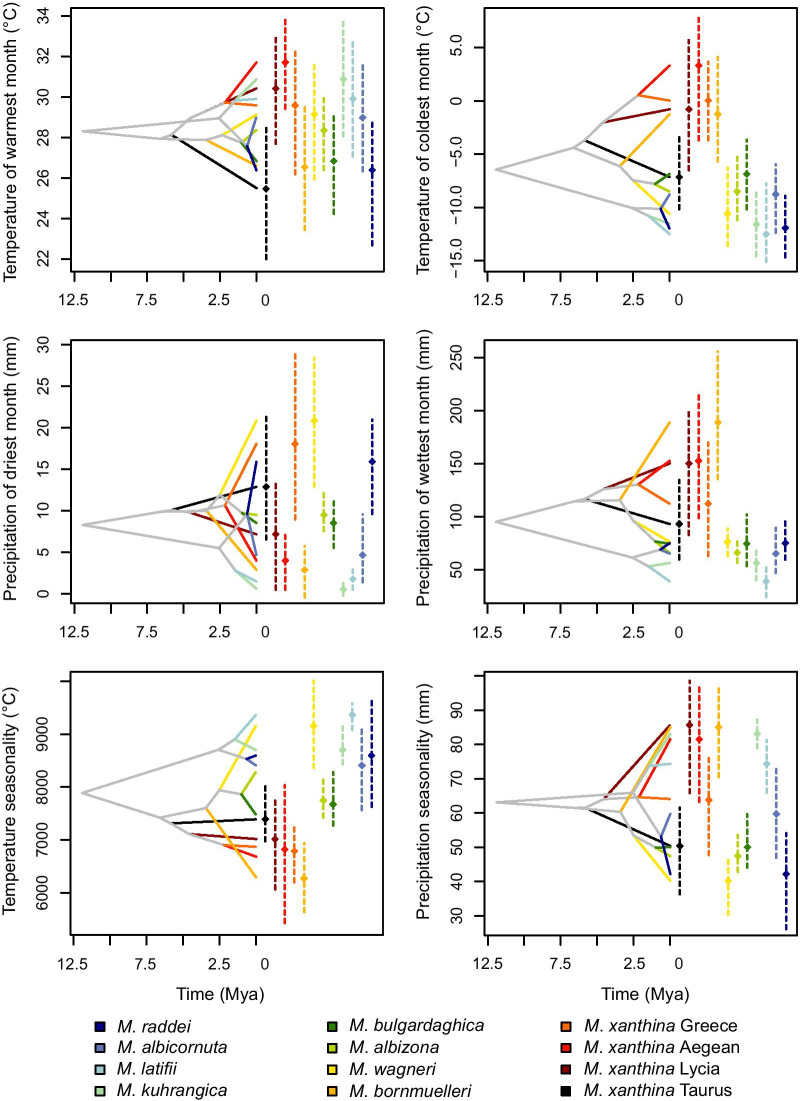
Inferred history of the evolution of environmental tolerances of six climatic variables within the mountain viper species based on a BEAST chronogram. Internal nodes denote the mean of climatic tolerances as estimated for the most recent common ancestor of the related extant taxa. Vertical bars and point marks show the 80% central density of environmental tolerance for each extant taxon and the related mean values, respectively. Lines and points are coloured according to clades defined in Fig. 1

The ancestral niche occupancy profiles (Fig. 3) provided evidence of both phylogenetic niche conservatism and niche divergence. The most noticeable phylogenetic niche conservatism was seen for *M. latifii* and *M. kuhrangica* with respect to almost all of the six climatic variables, and this is surprising considering the large geographic distance between their ranges. This pattern was also found for *M. albizona* and *M. bulgardaghica*, however, they are geographically in close proximity. Within the Raddei complex, *M. latifii*, *M. kuhrangica*, and *M. albicornuta* showed similar environmental affinities, while *M. raddei* revealed evidence of divergent evolution, particularly under extreme warm and dry climatic conditions. The most obvious phylogenetic niche divergence was seen for *M. bornmuelleri* and coastal lineages of *M. xanthina* (*M. xanthina* Lycia, *M. xanthina* Aegean, *M. xanthina* Greece), which have evolved in different directions compared to their close sister taxa. Notwithstanding, they showed between-group convergent evolution, particularly with respect to the precipitation variables.

### Reconstructing biogeographic history

Ancestral range estimation analyses revealed that based on the likelihood-ratio test comparing six biogeographic models, the DIVALIKE model is supported best (See Additional file 1: Table S3 for complete biogeographic comparison outputs). The resulting parameters of the DIVALIKE model included the anagenetic dispersal rate (*d*) of 0 and a high cladogenetic dispersal rate (*j*) of 0.407. Reconstruction of the biogeographic history of mountain vipers using a BEAST chronogram in BIOGEOBEARS is illustrated in Fig. 1, showing the area or combined areas with the highest probability for each node. The ancestral range estimations showed that the most probable ancestral area for extant species of the Xanthina complex is highly likely split by a vicariance event between three areas: the Taurus Mountains (gray polygon in Fig. 1), the Levant (orange polygon in Fig. 1), and the Anatolian Diagonal (green polygon in Fig. 1). A striking biogeographic history was revealed in the *M. xanthina* clade, where coastal lineages (*M. xanthina* Lycia, *M. xanthina* Aegean, and *M. xanthina* Greece) dispersed toward Mediterranean coastal habitats in southwest and western Turkey from a mountain-dwelling ancestor. Reconstructions within the Bornmuelleri clade indicated two subsequent dispersal events from the ancestor to the Levant, i.e. *M. bornmuelleri*, and to the northeastern Turkey, i.e. *M. wagneri*, followed by a vicariance in Anatolian Diagonal between *M. bulgardaghica* and *M. albizona*. Supporting our results, Stümpel et al. [26] estimated that the geographic location of the ancestor of the Xanthina complex, including the Bornmuelleri clade, occurred in Anatolian Taurus Mountains in southern Turkey. The estimated ancestral areas for the Raddei basal node seemed to have diverged rapidly by repeated vicariance events between three areas of the Caucasus—Kurdistan, the Alborz Mountains, and the Zagros Mountains.

### Ancestral state estimation

**Fig. 4:**
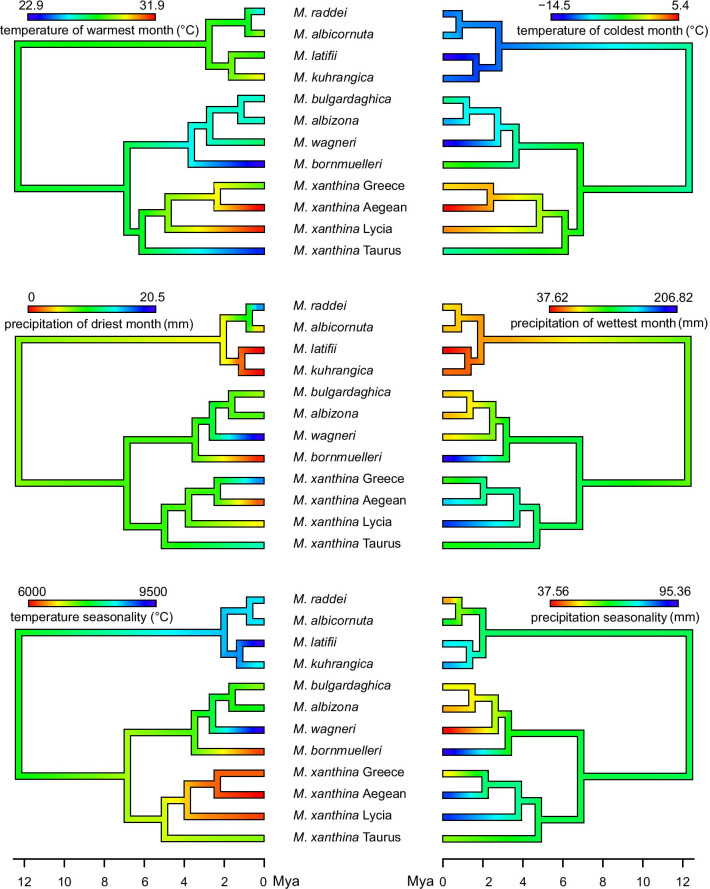
Ancestral state estimation of climatic variables for mountain vipers reconstructed based on a BEAST chronogram using the *contMap* function in the phytools R package

We found phylogenetic signals under both Pagel’s λ (*P* < 0.01) and Blomberg’s K (*P* < 0.05) for four of the six climatic variables: temperature seasonality, maximum temperature of the warmest month, minimum temperature of the coldest month, and precipitation of the driest month (Table 1). Based on the AICc statistic, the Brownian motion model showed the best fit of trait evolution for these bioclimatic variables, and precipitation of the wettest month and precipitation seasonality followed the white noise evolutionary model (Additional file 1: Table S3). Maximum likelihood ancestral state estimation (Fig. 4) based on the BM model indicated a mix of phylogenetic niche conservatism and divergent trait evolution (here climatic variables) in mountain vipers. Similar to the niche evolution chronogram, the ancestral state phenogram also confirmed that coastal lineages of *M. xanthina* (*M. xanthina* Lycia, *M. xanthina* Aegean, *M. xanthina* Greece) have highly likely diverged from a mountain-dwelling population. Relatively firm phylogenetic conservatism was seen in species of the Raddei complex. However, in this group, *M. latifii* in comparison to its ancestor has evolved toward extreme environmental conditions with a colder climate and higher temperature seasonality. Similarly, in the Bornmuelleri clade, *M. bornmuelleri* and *M. wagneri* have evolved divergently toward opposite climatic conditions compared to their most recent common ancestor.

**Table 1:** Statistical evidence of the phylogenetic signal in the six climatic variables influencing mountain viper range dynamics

	Pagel’s λ		Blomberg’s K	
	λ	*P*-value	K	*P*-value
Maximum temperature of warmest month	1.032	**0.006**	0.885	**0.007**
Minimum temperature of coldest month	1.028	**0.008**	0.998	**0.003**
Temperature seasonality	1.056	**0.003**	0.807	**0.008**
Precipitation of driest month	1.054	**0.009**	0.767	**0.011**
Precipitation of wettest month	6.98e−05	1	0.243	0.624
Precipitation seasonality	6.98e−05	1	0.359	0.257

## Discussion

The taxonomic status of mountain vipers of the genus *Montivipera* has long been the subject of debate among many herpetologists and taxonomists. All species in this group experienced a complex taxonomic history. For example, Mertens et al. [32] resolved *M. bornmuelleri* as *Vipera lebetina bornmuelleri*, a subspecies of the later *Macrovipera* genus. Sigg et al. [33] suggested that this species was *Vipera xanthina* when the *Montivipera* genus had not yet been introduced, and many species of this genus along *Macrovipera* and *Daboia* were considered to be monophyletic groups under the genus *Vipera*. Nilson and Andrén [34] first suggested *Montivipera* and *Macrovipera* as subgenera of *Vipera*, and Joger [35] later resurrected them as different genera. The present research, due to the use of a complete genetic dataset of mountain vipers, can be considered as the most comprehensive study of the phylogeny and biogeography of the genus *Montivipera*. Since our phylogenetic inference is based on only mitochondrial data we acknowledge that it might raise levels of uncertainty in species tree reconstruction as reliance on mitochondrial data can have ramifications for both evolutionary inference and taxonomy [36]. Indeed, male-biased dispersal, i.e. gene-flow, might violate inferences of mitochondrial markers especially in continuous species [37] or even in snakes [36, 38]. Although mountain vipers are extremely range-limited with low movement abilities, any interpretation on their taxonomic delimitation using only mitochondrial data should be taken cautiously.

The results of this study highlight the role of mountain ranges in producing and maintaining diversity in the eastern Mediterranean region. Particularly during times of orogeny, mountains facilitate geographic isolation and formation of allopatric speciation [25], a pattern that has been reported for a number of species groups [21, 22, 24, 39]. Based on the inferred phylogeny, the three monophyletic clades Xanthina, Bornmuelleri, and Raddei were verified. The first split in the ancestor of mountain vipers occurred around 12.18 Mya, which coincides with the Middle-to-Late Miocene. Similarly, Stümpel et al. [26] estimated the divergence time between the Xanthina and Raddei complex around 12.3 Mya. This period overlaps with widespread tectonic changes in the Near and Middle East, which include the uplift of the Irano-Anatolian mountains and disconnection between the Tethys Sea and Indian Ocean [40]. With continued tectonic changes in the region and the gradual disappearance of the Tethys Sea and formation of the Para-Tethys Sea in the Late Miocene, two clades, Xanthina in the west and Bornmuelleri in the central east of Anatolia, were separated. The increase of elevation to 1.5–2 km a.s.l in this period [41] likely stimulated major changes in climatic conditions and repetitive altitudinal shifts in biotic communities of the region, and highly likely, led to changes in the ecological niches and tolerances of *Montivipera* species. Generally, our estimated divergence times in the *Montivipera* genus are consistent with those of previous studies, for example see Stümpel et al. [26] and Behrooz et al. [30].

A considerable divergence in mountain vipers is the split of coastal vipers from their mountain-dwelling ancestor around 4.6 Mya, confirmed by niche evolution and ancestral range estimations. A possible reason for this divergence could be the absence of *Macrovipera lebetina* in the Mediterranean coastal ranges [31], a dominant competitor of *Montivipera* that occupies lower-elevated habitats in co-existing ranges. Overall, in the Bornmuelleri and Raddei clades, all the divergence events have occurred during Pliocene and Pleistocene climatic oscillations. Avise et al. [42] reported 57% of speciation in herpetofauna as a consequence of Pleistocene glaciations. Likewise, Veith et al. [43] and Plötner et al. [44] argued that the highest rates of speciation in the amphibians of Anatolia coincides with this time era. As suggested by BIOGEOBEARS range estimations, a progressive dispersal-vicariance provoked by the repeated fragmentation-reconnection during climatic cycles in this time period derived vicariance in different populations of mountain vipers and the foundation of allopatric speciation. This expedited phylogenetic niche conservatism, especially in *M. latifii, M. kuhrangica*, *M. xanthina* Taurus, *M. albizona*, and *M. bulgardaghica*, as supported by niche evolution and ancestral state estimation, emphasises the classic model of niche conservatism: conservation of niches after allopatric speciation in sister taxa [7, 20]. Here, allopatric speciation spatially overlaps with the floral glaciation refuges in the Taurus, Levant, and Anatolian Diagonal documented by Médail and Diadema [45].

Overall, the results of this research reveal a complex mixture of niche evolution in mountain vipers in terms of phylogenetic niche conservatism and divergence. The extreme divergent evolution in the climatic tolerance of mountain vipers was seen in the species of the Bornmuelleri clade. While *M. wagneri* and *M. latifii* have convergently evolved toward areas with the highest temperature seasonality and negative extremes of temperature, *M. bornmuelleri* has adapted to humid ranges with the lowest temperature seasonality. Consistently, temperature seasonality showed the strongest phylogenetic signal and was ranked among the most important variables contributing to the current distribution of mountain vipers (Additional file 1: Table S2). This consistency is one line of evidence that the Mediterranean ranges of Irano-Anatolia, along with Irano-Turanian, are characterised by their continentality and high temperature seasonality [46]. A similar pattern has also been revealed by divergent climatic tolerance in lizards of the genus *Timon* occupying opposite sides of the Mediterranean [47]. It should be naoted that the inherent low sample size of the species occurrence points might increase uncertainty in the predicted ENMs niche evolution analyses of the species. Pooling data of closely related taxa into one set or simply removing those with low sample sizes were the options we could consider in this study. The former might end with the inflation in the predicted niche of the species [48] which in turn might generalise distinct processes that mountain viper have been evolved through. Interestingly, those species with the lowest distribution area and the least number of occurrence points, e.g. *M. kuhrangica*, *M. latifii*, and *M. bornmuelleri*, are among the few mountain vipers that are classified as true species from a taxonomic viewpoint. As a consequence, we chose not to remove them from the analyses as they each show a noteworthy status in the phylogenetic and niche modelling analyses. For the ENM analysis, we chose MaxEnt as this method has been reported the most capable in producing useful results with small sample sizes, even less than 25 occurrences [48].

We considered the rate of niche evolution as the difference between the occupied niche of sister taxa and the projected niche of their most recent ancestor (y axes of Fig. 3) given the estimated divergence time (x axes of Fig. 3). Our results indicate that species of the Raddei complex have experienced slower rates of niche evolution, and given PNO and ancestral state reconstruction, have adapted to similar gradients of environmental space. Small population sizes, restricted distributions, and low genetic and haplotype diversity [30, 49] in the species of this complex are highly threatening and could negatively influence their adaptive capacities. Species with slower evolutionary changes in their niches compared to those experiencing rapid niche evolution are more vulnerable to demographic decline and population fluctuation [6, 50]. Particularly, niche conservatism in mountain vipers leads to lower resistance and adaptive capacity toward environmental changes [6]. Conversely, species with higher genetic diversity often have had rapid evolutionary rates in the past, and presently have broader environmental tolerance and niche breath, thus occupying wider geographic ranges [51, 52].

Generally, our results demonstrate that severe environmental changes in the past due to rapid orogeny and striking topographic heterogeneity in the mountain belts of Irano-Anatolia and the Caucasus have forced mountain vipers to experience a variety of niche evolution and diversification patterns. Due to their tendency towards niche conservatism and their low genetic diversity, these species are being forced to move their ranges or become extinct as the environment changes. Altitudinal range shift in mountain vipers has been demonstrated as a response to climatic changes [49]. This results in greater isolation and limited gene flow, intensifying extinction risk in these sky-island taxa. Population decline, isolated distributions, low genetic diversity, and low adaptive capacity create challenges for the conservation of mountain vipers. According to Fraser and Bernatchez [53], mountain-dwelling species with isolated distribution and limited gene flow are high-priority evolutionary distinct conservation units. The concept of evolutionary significant units (ESUs) and/or cryptic species can be considered as a consolidation of evolutionary biology, taxonomy, and conservation planning. While identifying evolutionary distinct taxa and their geographic distribution patterns is the cornerstone of biogeography and speciation [2], focus on these species as evolutionary ESUs and the existence of threatened taxa within cryptic complexes reflect the necessity for conservation considerations [3]. In this regard, focus on ecological diversification along with the use of ENMs facilitate understanding speciation process as well as taxonomic delimitations [12, 13, 54]. Here by using mountain vipers as striking models of neo-endemism in mountains of Eastern Mediterranean we bring together biogeographic, taxonomic, and conservation objectives into one set. Due to the unique biogeographic history and limited adaptive capacity we suggest that all the isolated populations of mountain vipers thus could be considered as ESUs requiring immediate conservation attention. Establishing conservation zones and adaptive conservation strategies such as assisted migration, and reducing threats such as overgrazing, habitat destruction, and over-collection for anti-venom production, are among the most important potential conservation strategies.

## Conclusion

Mountain vipers of the genus *Montivipera* are interesting examples of ecological species on the plateaus of Iran, Anatolia, and the Caucasus. On the one hand, their entire global reach is limited to this area, on the other hand, a high rate of neo-endemism has occurred in this genus. The combination of information obtained from phylogenetic reconstruction and ENM allowed unveiling the niche evolution of the species during diversification processes. Our results demonstrate that mountain vipers have been isolated from their common ancestors through common dispersal—vicariance processes, especially during the Pliocene–Pleistocene glacial fluctuations. The orogenic system of the region has additionally forced the genus to experience a high and complex diversity of ecological niche evolution of the species. In general, the species of Bornmuelleri clade have experienced the highest rate of evolutionary divergence toward climatic gradients while in the Raddei clade the species have had slow and convergence evolution through the climatic niche conditions. In conclusion, our study reveals limited evolutionary flexibility, and thus, adaptive capacity in mountain vipers especially for those distributed across mountains of Alborz and Zagros in Iran.

## Materials and methods

### Data collection

Species occurrence points for ENM analysis were recorded during tissue sampling of direct field surveys conducted by three authors of this study (M.A., R.B., and F.G., n = 105), and also obtained from other herpetologists’ field surveys (n = 62), existing databases (i.e. HerpNet, n = 10), and scientific publications (n = 143). Particularly, the study by Mebert et al. [55] has noticeably provided improved distribution data of the *Montivipera* of central-eastern Anatolia. Overall, we obtained 320 presence points covering the entire distribution of mountain vipers’ (Fig. 1) and ranging from 12 occurrences for *M. kuhrangica* to 51 localities for *M. albizona*.

In order to reconstruct a dated molecular tree, we downloaded 177 mitochondrial sequences from NCBI GenBank generated by previous studies. Specifically, our dataset comprised 82 sequences of cytochrome b (Cyt-b, 1061 bp) and ND4 (640 bp) generated by Behrooz et al. [30] for the Raddei complex. Additionally, 95 genetic sequences of three mitochondrial genes (Cyt-b, COX1, and ND5) produced by Stümpel et al. [26] for both Xanthina and Raddei complexes were downloaded. Details of the number of sequences and presence points are shown in Additional file 1: Table S1.

### Phylogenetic analysis and estimation of divergence times

As congruence plays an important role in crucial phylogenetic analyses based on multi-locus data [56], we tested congruence among four mitochondrial genes using Shimodaira–Hasegawa (SH) test. Results showed congruence among Cytb, COX1, and ND5, and accordingly a dataset matrix with 2488 bp (Cytb 1061 bp, COX1 824 bp, and ND5 602 bp) was considered for conducting the phylogenetic analysis. All sequences were aligned using ClustalW, implemented in MEGA v.6 [57]. We then concatenated the genetic sequences to one multi-locus dataset ranging from five sequences for *M. bornmuelleri* to 53 sequences for *M. albicornuta* (see Additional file 1). Best models of sequence evolution based on a partitioning scheme were chosen using a “greedy” algorithm and the Bayesian Information Criterion (BIC) in PartitionFinder 1.1.1 [58].

Divergence dates among 12 main lineages of *Montivipera* sp, discovered also in previous studies (Stümpel et al. [26], were calculated using BEAST 1.8.0 [59]. Molecular clock was carried out with two calibration points recommended by Stümpel et al. [26] including (i) the divergence between *Macrovipera* and *Montivipera* at ~ 15.5 Mya using a normal distribution with SD of 0.5, and (ii) the split between species of *M. xanthina* and *M. raddei* complexes using a normal distribution with a mean of 12.6 Mya and a standard deviation of 1.2 Mya. Accordingly, additional sequences of Cytb for two species of *Macrovipera*, including Levantine viper *Macrovopera lebetina* and Razi’s viper *Macrovipera razii*, were obtained from GenBank and incorporated to our dataset. Two independent runs were performed at 200 million generations and sampled every 10,000 generations, and omitting 25% as burn-in. Tracer 1.5 [60] was used for evaluating acceptable levels of MCMC chain mixing, and for checking convergence and effective sample sizes for all parameters. LOGCOMBINER V. 1.8.2 was applied to combine trees and log files. Finally, the maximum clade credibility tree with mean ages was summarised in TREEANNOTATOR v1.8.0 with a PP limit of 0.95.

### Ecological niche evolution

We used the ancestral niche occupancy reconstruction approach proposed by Evans et al. [61] to reconstruct patterns of niche evolution within mountain vipers. This method integrates each species’ potential distribution with the dated, ultrametric tree to explore environmental niche evolution by constructing predicted niche occupancy (PNO) profiles [61]. We limited ancestral niche reconstructions to a single terminal per species by pruning replicate branches off the *Montivipera* BEAST tree using the *drop.tip* function in the R package ape [62].

Species’ potential distribution was modelled by using the maximum entropy (MaxEnt) method [63] and six climatic variables representing climate extremes and seasonal variability in the region, including maximum temperature of the warmest month (°C), minimum temperature of the coldest month (°C), temperature seasonality, precipitation of the driest month (mm), precipitation of the wettest month (mm), and precipitation seasonality. By reflecting adaptation to extreme climatic conditions, these variables represent important environmental constraints on species distributions, niche evolution, and adaptability [64]. Also, this selection avoids collinearity problems as the pairwise correlation fulfils |r|< 0.7. Data on climatic variables with a spatial resolution of 30 arc-second were obtained from the WorldClim database [65]. We used MaxEnt model as this method has proven to obtain more reliable results with small sample sizes even lower than 25 occurrences [48]. Additionally, we adopted two methods to reduce the uncertainty caused by the inherent low sample size of mountain vipers’ presence data. First, to cope with the negative impacts of spatial autocorrelation (SAC), we set the minimum distance between species presence to 1 km. Second, for each species we repeated MaxEnt modelling 10 times based on a bootstrap procedure. The predictive performance of models was evaluated based on the area under the curve (AUC) of receiver operating characteristic (ROC) plots. Because of the drawbacks of AUC to measure the predictive performance of distribution models [66], we also calculated the true skill statistic (TSS) as a measure of the model’s classification accuracy. We considered the minimum suitability at the presence points as a suitability threshold to calculate TSS.

To reconstruct ancestral niche occupancy profiles, we combined each species’ potential distribution at a given grid cell in the study area with the corresponding scores of the six original climatic variables to create a vector of probabilities per binned climatic values (here we calculated it for 100 intervals). To generate profiles of predicted niche occupancy (PNO), the map of each climatic variable was converted to a histogram of 100 equal-interval bins, and the total MaxEnt raw probabilities in each bin were calculated for each species. By using this method, instead of extracting climatic data from species localities to reconstruct ancestral climatic tolerances, we linked habitat suitability with bins of climatic variables [61]. We then reconstructed the climatic tolerances of ancestral nodes using the PNO profiles under the assumption of Brownian motion evolution. To do so, the PNO profiles were resampled 1000 times, and the ancestral character reconstructions were performed using the generalised least squares method [for more detail see 62]. The ancestral niche reconstructions were computed using the package phyloclim [67].

### Ancestral range estimation

We inferred ancestral ranges and the biogeographic history of mountain vipers based on the pruned divergence time tree and three models of biogeographical range expansion, including Dispersal-Extinction-Cladogenesis (DEC), Dispersal-Vicariance (DIVA-like), and Bayesian inference (BAYAREA-like) models, all using the BioGeoBEARS package [68] in the R environment. Using these models enabled us to explore different possibilities of dispersal, vicariance, and extinction. We also incorporated a founder-event parameter (+ J) in the analysis allowing for cladogenic dispersal outside of the parental areas, i.e., jump speciation [69]. We divided mountain vipers into eight biogeographic areas: (1) Taurus Mountains, (2) Mediterranean coasts and islands, (3) the Levant, (4) Anatolian Diagonal, (5) Northeast Anatolia, (6) South Caucasus—Kurdistan, (7) Alborz Mountains, and (8) South Zagros Mountains. These biogeographic areas were selected given the topology of mountain vipers in their phylogenetic time tree as well as the physiography of the selected mountain patches in the region. Specifically, these biogeographic areas are geographically isolated except for the Anatolian Diagonal and Northeast Anatolia, for which the reason for considering them as different groups was that we additionally intended to reveal the species-specific biogeographic history of mountain vipers. To facilitate clear estimations, we allowed inferred ancestral ranges to occupy up to two areas, with relative probabilities of dispersal and the possibility to disperse in four time-slices (0–0.40, 0.40–4, 4–8, and 8–11.5 Mya) considering divergence times within the complex. Likelihood estimates of three biogeographic models and their modified J types were compared using the Akaike Information Criterion (AIC) to identify the best-fit model.

### Ancestral state estimation

We illustrated the ancestral state estimations of six climatic variables to assess phylogenetic niche conservatism (PNC) in mountain vipers [7]. First, phylogenetic signals were evaluated to examine whether similarity in lineage traits (here, the climatic variables) is correlated with phylogenetic relationships. As input data, we averaged each of the six climatic variables extracted from each of the species presence points. We tested for phylogenetic signals in the six climatic variables based on Blomberg’s K [70] and Pagel’s λ [71] using the *phylosig* function of package phytools [72]. We then tested for PNC under three models of trait evolution: Brownian motion (BM), Ornstein–Uhlenbeck (OU), and white noise (WN) using the *fitContinuous* function of package geiger [73]. Selection of the WN model indicates that traits are evolving independently without phylogenetic signal; BM assumes that the correlation structure among trait values is proportional to the extent of shared ancestry for pairs of species, and OU implies stabilising selection over time. The best model was ranked based on the AICc. Finally, a maximum likelihood framework was used to estimate the ancestral state of internal nodes under the selected evolutionary model, and the inferred states were interpolated along the edges of each branch based on Felsenstein’s equation [74] using the *contMap* function of package phytools [75].

## Supplementary Information

**Additional file 1: Table S1.** Number of occurrence points and mitochondrial sequences used to reconstruct phylogenetic time tree and perform ecological niche modelling of mountain vipers.** Table S2.** Average percent contribution of six climatic variables, area under the receiver operating characteristic curve (AUC) and true statistics skill (TSS) of the ecological niche models performed for mountain vipers of the genus Montivipera in the eastern Mediterranean region.** Table S3.** Results of assessing the best evolutionary models of bioclimatic variables affecting Montivipera based on AICc statistics, using the BEAST chronogram and the fitContinuous function of geiger package. Bold values show the best model. AICc: Akaike Information Criterion for small sample-sized data, Ll: Log-likelihood.** Table S4.** List of samples, geographical origin, id and accession numbers for genes of the genus Montivipera and out-groups used in the phylogeographic and molecular dating analyses.** Fig. S1.** The Bayesian phylogeny tree reconstructed from the 177 sequences of mountain vipers (Montivipera). The tree was reconstructed based on a partitioned analysis with three Mitochondrial genes (CYTB, COX1, ND5). Values above and below the branches are the posterior probabilities and mean age estimates, respectively. Colors of the branches corresponds to the geographic origin of the species as shown in Fig. 1.

## Data Availability

The XML file used to reconstruct phylogenetic analysis, the R code, and the input data for the niche evolution and the ancestral estimation analysis are available at: https://github.com/mahmadi-iut/Montivipera-niche-evolution.git
