# Supplementary material for: The legacy of Eastern Mediterranean mountain uplifts: rapid disparity of phylogenetic niche conservatism and divergence in mountain vipers
Source: BMC Ecol Evol. 2021 Jun 22;21:130. doi: 10.1186/s12862-021-01863-0 (PMC8220690; doi:10.1186/s12862-021-01863-0)
Supplement: Supplementary file 1 — Additional file 1: Table S1. Number of occurrence points and mitochondrial sequences used to reconstruct phylogenetic time tree and perform ecological niche modelling of mountain vipers. Table S2. Average percent contribution of six climatic variables, area under the receiver operating characteristic curve (AUC) and true statistics skill (TSS) of the ecological niche models performed for mountain vipers of the genus Montivipera in the eastern Mediterranean region. Table S3. Results of assessing the best evolutionary models of bioclimatic variables affecting Montivipera based on AICc statistics, using the BEAST chronogram and the fitContinuous function of geiger package. Bold values show the best model. AICc: Akaike Information Criterion for small sample-sized data, Ll: Log-likelihood. Table S4. List of samples, geographical origin, id and accession numbers for genes of the genus Montivipera and out-groups used in the phylogeographic and molecular dating analyses. Fig. S1. The Bayesian phylogeny tree reconstructed from the 177 sequences of mountain vipers (Montivipera). The tree was reconstructed based on a partitioned analysis with three Mitochondrial genes (CYTB, COX1, ND5). Values above and below the branches are the posterior probabilities and mean age estimates, respectively. Colors of the branches corresponds to the geographic origin of the species as shown in Fig. 1. [file 12862_2021_1863_MOESM1_ESM.docx]

**Table S1.** Number of occurrence points and mitochondrial sequences used to reconstruct phylogenetic time tree and perform ecological niche modelling of mountain vipers.

|  | Number of Occurrence points | Total number of sequences |
| --- | --- | --- |
| *M. raddei* | 45 | 65 |
| *M. albicornuta* | 24 | 20 |
| *M. latifii* | 18 | 18 |
| *M. kuhrangica* | 12 | 7 |
| *M. bulgardaghica* | 33 | 8 |
| *M. albizona* | 51 | 7 |
| *M. wagneri* | 36 | 12 |
| *M. bornmuelleri* | 22 | 5 |
| *M. xanthina* Greece | 15 | 16 |
| *M. xanthina* Aegean | 16 | 7 |
| *M. xanthina* Lycia | 20 | 7 |
| *M xanthina* Taurus | 28 | 5 |

**Table S2**. Average percent contribution of six climatic variables, area under the receiver operating characteristic curve (AUC) and true statistics skill (TSS) of the ecological niche models performed for mountain vipers of the genus *Montivipera* in the eastern Mediterranean region.

|  | Bio4 | Bio5 | Bio6 | Bio13 | Bio14 | Bio15 | AUC | TSS |
| --- | --- | --- | --- | --- | --- | --- | --- | --- |
| *M. raddei* | 29.6 | 1.7 | 45.2 | 6.2 | 13.1 | 4.3 | 0.91 | 0.89 |
| *M. albicornuta* | 6.4 | 1.5 | 68.7 | 1.5 | 20.9 | 1 | 0.90 | 0.88 |
| *M. latifii* | 29.6 | 1 | 61.7 | 3.4 | 4.4 | 0 | 0.92 | 0.93 |
| *M. kuhrangica* | 31.6 | 1 | 59.7 | 4.5 | 3.3 | 0 | 0.94 | 0.94 |
| *M. bulgardaghica* | 17 | 2.7 | 18.7 | 5.4 | 40.4 | 15.8 | 0.92 | 0.9 |
| *M. albizona* | 18.4 | 4.2 | 39.3 | 5.6 | 28.1 | 4.4 | 0.90 | 0.88 |
| *M. wagneri* | 44 | 2 | 36.3 | 4.1 | 0 | 13.6 | 0.89 | 0.90 |
| *M. bornmuelleri* | 0 | 32.3 | 1.6 | 41.8 | 10.6 | 13.8 | 0.95 | 0.94 |
| *M. xanthina* Greece | 45.1 | 0.6 | 29.7 | 6.3 | 8.5 | 9.7 | 0.88 | 0.89 |
| *M. xanthina* Aegean | 11.9 | 0 | 12.4 | 53.8 | 21.9 | 0 | 0.90 | 0.92 |
| *M. xanthina* Lycia | 0.7 | 0 | 0.7 | 51.9 | 17 | 29.6 | 0.94 | 0.95 |
| *M xanthina* Taurus | 19.8 | 38.2 | 15.5 | 23.7 | 2.3 | 0.6 | 0.94 | 0.95 |

**Table S3.** Results of assessing the best evolutionary models of bioclimatic variables affecting *Montivipera* based on AICc statistics, using the BEAST chronogram and the *fitContinuous* function of geiger package. Bold values show the best model. AICc: Akaike Information Criterion for small sample-sized data, Ll: Log-likelihood.

| Bioclimatic variables | BM | | OU | | WN | |
| --- | --- | --- | --- | --- | --- | --- |
|  | AICc | Ll | AICc | Ll | AICc | Ll |
| Maximum temperature of warmest month | **122.04** | **-58.35** | 122.57 | -56.78 | 125.02 | -59.84 |
| Minimum temperature of coldest month | **132.61** | **-63.64** | 136.22 | -63.61 | 139.501 | -67.08 |
| Temperature seasonality | **205.43** | **-100** | 208.56 | -99.78 | 209.77 | -102.2 |
| Precipitation of driest month | **131.18** | **-62.92** | 134.41 | -62.71 | 135.93 | -64.79 |
| Precipitation of wettest month | 96.43 | -45.55 | 90.07 | -40.53 | **86.34** | **-40.51** |
| Precipitation seasonality | 116.24 | -55.45 | 114.05 | -52.52 | **110.69** | **-52.27** |

**Table S4**. List of samples, geographical origin, id and accession numbers for genes of the genus *Montivipera* and out-groups used in the phylogeographic and molecular dating analyses.

| **#** | **Species** | **Locality** | **ID** | **Cytb** | **ND4** | **COI** | **ND5** |
| --- | --- | --- | --- | --- | --- | --- | --- |
| 1 | *Montivipera latifii* | Lar National Park, Iran | BEV.T7010 | MG021062 | MG041952 |  |  |
| 2 | *Montivipera latifii* | Lar National Park, Iran | BEV.T7011 | MG021063 | MG041953 |  |  |
| 3 | *Montivipera latifii* | Lar National Park, Iran | BEV.T7012 | MG021064 | MG041954 |  |  |
| 4 | *Montivipera latifii* | Lar National Park, Iran | BEV.T7013 | MG021065 | MG041955 |  |  |
| 5 | *Montivipera albicornuta* | Tarom, Iran | BEV.T7014 | MG021000 | MG041956 |  |  |
| 6 | *Montivipera latifii* | Lar National Park, Iran | BEV.T7015 | MG021066 | MG041957 |  |  |
| 7 | *Montivipera albicornuta* | Tarom, Iran | BEV.T7016 | MG021001 | MG041958 |  |  |
| 8 | *Montivipera latifii* | Lar National Park, Iran | BEV.T7017 | MG021067 | MG041959 |  |  |
| 9 | *Montivipera albicornuta* | Tarom, Iran | BEV.T7018 | MG021002 | MG041960 |  |  |
| 10 | *Montivipera latifii* | Lar National Park, Iran | BEV.T7019 | MG021068 | MG041961 |  |  |
| 11 | *Montivipera albicornuta* | Tarom, Iran | BEV.T7020 | MG021003 | MG041962 |  |  |
| 12 | *Montivipera latifii* | Lar National Park, Iran | BEV.T7021 | MG021069 | MG041963 |  |  |
| 13 | *Montivipera latifii* | Lar National Park, Iran | BEV.T7022 | MG021070 | MG041964 |  |  |
| 14 | *Montivipera latifii* | Lar National Park, Iran | BEV.T7023 | MG021070 | MG041965 |  |  |
| 15 | *Montivipera latifii* | Lar National Park, Iran | BEV.T7024 | MG021072 | MG041966 |  |  |
| 16 | *Montivipera latifii* | Lar National Park, Iran | BEV.T7025 | MG021073 | MG041967 |  |  |
| 17 | *Montivipera albicornuta* | Tarom, Iran | BEV.T7026 | MG021004 | MG041968 |  |  |
| 18 | *Montivipera latifii* | Lar National Park, Iran | BEV.T7027 | MG021074 | MG041969 |  |  |
| 19 | *Montivipera albicornuta* | Tarom, Iran | BEV.T7028 | MG021005 | MG041970 |  |  |
| 20 | *Montivipera albicornuta* | Tarom, Iran | BEV.T7029 | MG021006 | MG041971 |  |  |
| 21 | *Montivipera raddei* | Qorveh, Iran | BEV.T7030 | MG021007 | MG041972 |  |  |
| 22 | *Montivipera raddei* | Qorveh, Iran | BEV.T7031 | MG021008 | MG041973 |  |  |
| 23 | *Montivipera raddei* | Qorveh, Iran | BEV.T7032 | MG021009 | MG041974 |  |  |
| 24 | *Montivipera raddei* | Qorveh, Iran | BEV.T7033 | MG021010 | MG041975 |  |  |
| 25 | *Montivipera raddei* | Qorveh, Iran | BEV.T7034 | MG021011 | MG041976 |  |  |
| 26 | *Montivipera kuhrangica* | Kuhrang, Iran | BEV.T7035 | MG021054 | MG041977 |  |  |
| 27 | *Montivipera raddei* | Qorveh, Iran | BEV.T7036 | MG021012 | MG041978 |  |  |
| 28 | *Montivipera raddei* | Qorveh, Iran | BEV.T7037 | MG021013 | MG041979 |  |  |
| 29 | *Montivipera raddei* | Maku, Iran | BEV.T7039 | MG020984 | MG041980 |  |  |
| 30 | *Montivipera raddei* | Maku, Iran | BEV.T7040 | MG020985 | MG041981 |  |  |
| 31 | *Montivipera raddei* | Maku, Iran | BEV.T7041 | MG020986 | MG041982 |  |  |
| 32 | *Montivipera raddei* | Maku, Iran | BEV.T7042 | MG020987 | MG041983 |  |  |
| 33 | *Montivipera raddei* | Tekab, Iran | BEV.T7043 | MG021014 | MG041984 |  |  |
| 34 | *Montivipera raddei* | Qorveh, Iran | BEV.T7044 | MG021015 | MG041985 |  |  |
| 35 | *Montivipera raddei* | Qorveh, Iran | BEV.T7045 | MG021016 | MG041986 |  |  |
| 36 | *Montivipera raddei* | Qorveh, Iran | BEV.T7046 | MG021017 | MR041987 |  |  |
| 37 | *Montivipera raddei* | Qorveh, Iran | BEV.T7781 | MG021018 | MG041990 |  |  |
| 38 | *Montivipera raddei* | Qorveh, Iran | BEV.T7783 | MG021019 | MG041991 |  |  |
| 39 | *Montivipera raddei* | Qorveh, Iran | BEV.T7803 | MG021020 | MG041993 |  |  |
| 40 | *Montivipera raddei* | Qorveh, Iran | BEV.T7810 | MG021022 | MG041995 |  |  |
| 41 | *Montivipera raddei* | Qorveh, Iran | BEV.T7814 | MG021023 | MG041996 |  |  |
| 42 | *Montivipera albicornuta* | Zanjan, Iran | BEV.T7816 | MG021024 | MG041997 |  |  |
| 43 | *Montivipera albicornuta* | Zanjan, Iran | BEV.T7817 | MG021025 | MG041998 |  |  |
| 44 | *Montivipera raddei* | Zanjan, Iran | BEV.T7818 | MG021026 | MG041999 |  |  |
| 45 | *Montivipera raddei* | Zanjan, Iran | BEV.T7819 | MG021027 | MG042000 |  |  |
| 46 | *Montivipera raddei* | Zanjan, Iran | BEV.T7820 | MG021028 | MG042001 |  |  |
| 47 | *Montivipera albicornuta* | Zanjan, Iran | BEV.T7821 | MG021029 | MG042002 |  |  |
| 48 | *Montivipera raddei* | Zanjan, Iran | BEV.T7822 | MG021030 | MG042003 |  |  |
| 49 | *Montivipera raddei* | Sahand, Iran | BEV.T7823 | MG021031 | MG042004 |  |  |
| 50 | *Montivipera raddei* | Sahand, Iran | BEV.T7824 | MG021032 | MG042005 |  |  |
| 51 | *Montivipera raddei* | Sahand, Iran | BEV.T7825 | MG021033 | MG042006 |  |  |
| 52 | *Montivipera raddei* | Qotur, Iran | BEV.T7826 | MG020988 | MG042007 |  |  |
| 53 | *Montivipera raddei* | Qotur, Iran | BEV.T7827 | MG020989 | MG042008 |  |  |
| 54 | *Montivipera raddei* | Qotur, Iran | BEV.T7828 | MG020990 | MG042009 |  |  |
| 55 | *Montivipera raddei* | Qotur, Iran | BEV.T7829 | MG020991 | MG042010 |  |  |
| 56 | *Montivipera raddei* | Qotur, Iran | BEV.T7830 | MG020992 | MG042011 |  |  |
| 57 | *Montivipera raddei* | Maku, Iran | BEV.T7831 | MG020993 | MG042012 |  |  |
| 58 | *Montivipera raddei* | Maku, Iran | BEV.T7832 | MG020994 | MG042013 |  |  |
| 59 | *Montivipera raddei* | Maku, Iran | BEV.T7833 | MG020995 | MG042014 |  |  |
| 60 | *Montivipera raddei* | Maku, Iran | BEV.T7834 | MG020996 | MG042015 |  |  |
| 61 | *Montivipera raddei* | Maku, Iran | BEV.T7835 | MG020997 | MG042016 |  |  |
| 62 | *Montivipera raddei* | Maku, Iran | BEV.T7836 | MG020998 | MG042017 |  |  |
| 63 | *Montivipera raddei* | Maku, Iran | BEV.T7837 | MG020999 | MG042018 |  |  |
| 64 | *Montivipera raddei* | Tekab, Iran | BEV.T7838 | MG021034 | MG042019 |  |  |
| 65 | *Montivipera raddei* | Tekab, Iran | BEV.T7839 | MG021035 | MG042020 |  |  |
| 66 | *Montivipera raddei* | Tekab, Iran | BEV.T7840 | MG021036 | MG042021 |  |  |
| 67 | *Montivipera albicornuta* | Sarab, Iran | BEV.T7841 | MG021037 | MG042022 |  |  |
| 68 | *Montivipera albicornuta* | Sarab, Iran | BEV.T7842 | MG021038 | MG042023 |  |  |
| 69 | *Montivipera raddei* | Qorveh, Iran | BEV.T7876 | MG021039 | MG042024 |  |  |
| 70 | *Montivipera raddei* | Qorveh, Iran | BEV.T7884 | MG021041 | MG042026 |  |  |
| 71 | *Montivipera kuhrangica* | Kuhrang, Iran | BEV.T9282 | MG021055 | MG042028 |  |  |
| 72 | *Montivipera kuhrangica* | Tapeleh, Iran | BEV.T9285 | MG021056 | MG042029 |  |  |
| 73 | *Montivipera kuhrangica* | Tapeleh, Iran | BEV.T9286 | MG021057 | MG042030 |  |  |
| 74 | *Montivipera kuhrangica* | Tapeleh, Iran | BEV.T9290 | MG021059 | MG042034 |  |  |
| 75 | *Montivipera kuhrangica* | Kuhrang, Iran | BEV.T9291 | MG021060 | MG042035 |  |  |
| 76 | *Montivipera kuhrangica* | Kuhrang, Iran | BEV.T9292 | MG021061 | MG042036 |  |  |
| 77 | *Montivipera raddei* | Alvand, Iran | BEV.T9293 | MG021043 | MG042037 |  |  |
| 78 | *Montivipera raddei* | Alvand, Iran | BEV.T9294 | MG021044 | MG042038 |  |  |
| 79 | *Montivipera raddei* | Alvand, Iran | BEV.T9295 | MG021045 | MG042039 |  |  |
| 80 | *Montivipera raddei* | Tekab, Iran | BEV.T9297 | MG021047 | MG042041 |  |  |
| 81 | *Montivipera albicornuta* | Sarab, Iran | BEV.T9299 | MG021048 | MG042042 |  |  |
| 82 | *Montivipera raddei* | Bukan, Iran | BEV.T9300 | MG021049 | MG042043 |  |  |
| 83 | *Montivipera xanthina* | Loutros, Greece | BEV.T3656* | MG021075 | MG042050 |  |  |
| 84 | *Montivipera xanthina* | Loutros, Greece | BEV.T3669* | MG021076 | MG042051 |  |  |
| 85 | *Montivipera albicornuta* | Khān Shāy, Iran | ac1 | KX168711 |  | KX168812 | KX168914 |
| 86 | *Montivipera albicornuta* | Khān Shāy, Iran | ac2 | KX168714 |  | KX168815 | KX168917 |
| 87 | *Montivipera albicornuta* | Khān Shāy, Iran | ac3 | KX168715 |  | KX168816 | KX168918 |
| 88 | *Montivipera albicornuta* | Khān Shāy, Iran | ac4 | KX168716 |  | KX168817 | KX168919 |
| 89 | *Montivipera albicornuta* | Khān Shāy, Iran | ac5 | KX168717 |  | KX168818 | KX168920 |
| 90 | *Montivipera albicornuta* | Khān Shāy, Iran | ac6 | KX168718 |  | KX168819 | KX168921 |
| 91 | *Montivipera albicornuta* | Khān Shāy, Iran | ac7 | KX168719 |  | KX168820 | KX168922 |
| 92 | *Montivipera raddei* | Maku, Iran | ac11 | KX168753 |  | KX168854 | KX168963 |
| 93 | *Montivipera raddei* | Maku, Iran | ac12 | KX168754 |  | KX168855 | KX168964 |
| 94 | *Montivipera raddei* | Qotur, Iran | ac14 | KX168747 |  | KX168848 | KX168957 |
| 95 | *Montivipera albizona* | Kulmaç Dağları, Turkey | az3 | KX168722 |  | KX168823 | KX168925 |
| 96 | *Montivipera albizona* | Kulmaç Dağları, Turkey | az4 | KX168723 |  | KX168824 | KX168926 |
| 97 | *Montivipera albizona* | Kulmaç Dağları, Turkey | az5 | KX168724 |  | KX168825 | KX168927 |
| 98 | *Montivipera albizona* | Pιnarbaşι, Turkey | az6 | KX168725 |  | KX168826 | KX168928 |
| 99 | *Montivipera albizona* | Pιnarbaşι, Turkey | az7 | KX168726 |  | KX168827 | KX168929 |
| 100 | *Montivipera albizona* | Terra typica, Turkey | az9 | KX168727 |  | KX168828 | KX168930 |
| 101 | *Montivipera bulgardaghica* | Arslanköy, Turkey | bg3 | KX168738 |  | KX168839 | KX168942 |
| 102 | *Montivipera bulgardaghica* | Arslanköy, Turkey | bg4 | KX168739 |  | KX168840 | KX168943 |
| 103 | *Montivipera bulgardaghica* | Kar Boaz, Turkey | bg10 | KX168728 |  | KX168829 | KX168931 |
| 104 | *Montivipera bulgardaghica* | Arslanköy, Turkey | bg5 | KX168740 |  | KX168841 | KX168944 |
| 105 | *Montivipera bulgardaghica* | Bolkar Dağlarι, Turkey | bg12 | KX168735 |  | KX168836 | KX168939 |
| 106 | *Montivipera bulgardaghica* | Bolkar Dağlarι, Turkey | bg13 | KX168736 |  | KX168837 | KX168940 |
| 107 | *Montivipera bulgardaghica* | Bolkar Dağlarι, Turkey | bg16 | KX168737 |  | KX168838 | KX168941 |
| 108 | *Montivipera bornmuelleri* | Bsharri, Lebanon | bm1 | KX168730 |  | KX168831 | KX168933 |
| 109 | *Montivipera bornmuelleri* | Bsharri, Lebanon | bm6 | KX168733 |  | KX168834 | KX168936 |
| 110 | *Montivipera bornmuelleri* | Mt. Hermon, Lebanon | bm9 | KX168734 |  | KX168835 | KX168937 |
| 111 | *Montivipera bornmuelleri* | Mt. Hermon , Lebanon | bm10 | KX168731 |  | KX168832 | KX168934 |
| 112 | *Montivipera bornmuelleri* | Mt. Hermon, Lebanon | bm11 | KX168732 |  | KX168833 | KX168935 |
| 113 | *Montivipera latifii* | Lar valley, Iran | lt2 | KX168742 |  | KX168843 | KX168946 |
| 114 | *Montivipera latifii* | Lar valley, Iran | it5 | KX168743 |  | KX168844 | KX168947 |
| 115 | *Montivipera latifii* | Lar valley, Iran | lt6 | KX168744 |  | KX168845 | KX168948 |
| 116 | *Montivipera latifii* | Lar valley, Iran | lt7 | KX168745 |  | KX168846 | KX168949. |
| 117 | *Montivipera latifii* | Lar valley, Iran | lt8 | KX168746 |  | KX168847 | KX168950 |
| 118 | *Montivipera raddei* | Yüksekova, Turkey | rk1 | KX168748 |  | KX168849 | KX168958 |
| 119 | *Montivipera raddei* | Yüksekova, Turkey | rk3 | KX168750 |  | KX168851 | KX168960 |
| 120 | *Montivipera raddei* | Yüksekova, Turkey | rk7 | KX168751 |  | KX168852 | KX168961 |
| 121 | *Montivipera raddei* | Yüksekova, Turkey | rk8 | KX168752 |  | KX168853 | KX168962 |
| 122 | *Montivipera raddei* | Yüksekova, Turkey | rk10 | KX168749 |  | KX168850 | KX168959 |
| 123 | *Montivipera raddei* | Ararat, Turkey | rr1 | KX168755 |  | KX168856 | KX168965 |
| 124 | *Montivipera raddei* | Ararat, Turkey | rr2 | KX168757 |  | KX168859 | KX168969 |
| 125 | *Montivipera raddei* | Ararat, Turkey | rr3 | KX168759 |  | KX168861 | KX168971 |
| 126 | *Montivipera raddei* | Yeghegnadzor region, Saravan, Armenia | rr5 | KX168760 |  | KX168862 | KX168972 |
| 127 | *Montivipera raddei* | Kotayck region, Armenia | rr6 | KX168761 |  | KX168863 | KX168973 |
| 128 | *Montivipera raddei* | Digor, Armenia | rr7 | KX168762 |  | KX168864 | KX168974 |
| 129 | *Montivipera raddei* | Digor, Armenia | rr10 | KX168756 |  | KX168857 | KX168966 |
| 130 | *Montivipera raddei* | Van Gölü, Turkey | rr21 | KX168758 |  | KX168860 | KX168970 |
| 131 | *Montivipera albizona* | Göksun, Turkey | spec.1 |  |  | KX168830 | KX168932 |
| 132 | *Montivipera wagneri* | Aras Nehri, Turkey | wg3 | KX168770 |  | KX168872 | KX168982 |
| 133 | *Montivipera wagneri* | Aras Nehri, Turkey | wg4 | KX168771 |  | KX168873 | KX168983 |
| 134 | *Montivipera wagneri* | Aras Nehri, Turkey | wg5 | KX168772 |  | KX168874 | KX168984 |
| 135 | *Montivipera wagneri* | Aras Nehri, Turkey | wg6 | KX168773 |  | KX168875 | KX168985 |
| 136 | *Montivipera wagneri* | Aras Nehri, Turkey | wg7 | KX168774 |  | KX168876 | KX168986 |
| 137 | *Montivipera wagneri* | Aras Nehri, Turkey | wg8 | KX168775 |  | KX168877 | KX168987 |
| 138 | *Montivipera wagneri* | Sarιkamιş, Turkey | wg10 | KX168764 |  | KX168866 | KX168976 |
| 139 | *Montivipera wagneri* | Karakurt, Turkey | wg11 | KX168765 |  | KX168867 | KX168977 |
| 140 | *Montivipera wagneri* | Karakurt, Turkey | wg12 | KX168766 |  | KX168868 | KX168978 |
| 141 | *Montivipera wagneri* | Karakurt, Turkey | wg13 | KX168767 |  | KX168869 | KX168979 |
| 142 | *Montivipera wagneri* | Karakurt, Turkey | wg15 | KX168768 |  | KX168870 | KX168980 |
| 143 | *Montivipera wagneri* | Aras Nehri, Turkey | wg16 | KX168769 |  | KX168871 | KX168981 |
| 144 | *Montivipera xanthina* | Tekir Dağ, Turkey | xt1 | KX168777 |  | KX168879 | KX168989 |
| 145 | *Montivipera xanthina* | Tekir Dağ, Turkey | xt2 | KX168784 |  | KX168886 | KX168997 |
| 146 | *Montivipera xanthina* | Geyik Dağι, Turkey | xt4 | KX168800 |  | KX168902 | KX169013 |
| 147 | *Montivipera xanthina* | Geyik Dağι, Turkey | xt5 | KX168806 |  | KX168908 | KX169019 |
| 148 | *Montivipera xanthina* | Geyik Dağι, Turkey | xt6 | KX168809 |  | KX168911 | KX169022 |
| 149 | *Montivipera xanthina* | Isparta, Turkey | xt8 | KX168810 |  | KX168912 | KX169023 |
| 150 | *Montivipera xanthina* | Isparta, Turkey | xt9 | KX168811 |  | KX168913 | KX169024 |
| 151 | *Montivipera xanthina* | Izmir, Turkey | xt11 | KX168778 |  | KX168880 | KX168990 |
| 152 | *Montivipera bulgardaghica* | Bolkar Dağlarι, Turkey | xt12 | KX168741 |  | KX168842 | KX168945 |
| 153 | *Montivipera xanthina* | Selçuk, Turkey | xt15 | KX168779 |  | KX168881 | KX168991 |
| 154 | *Montivipera xanthina* | Selçuk, Turkey | xt16 | KX168780 |  | KX168882 | KX168992 |
| 155 | *Montivipera xanthina* | Selçuk, Turkey | xt17 | KX168781 |  | KX168883 | KX168993 |
| 156 | *Montivipera xanthina* | Selçuk, Turkey | xt18 | KX168782 |  | KX168884 | KX168994 |
| 157 | *Montivipera xanthina* | Selçuk, Turkey | xt19 | KX168783 |  | KX168885 | KX168995 |
| 158 | *Montivipera xanthina* | Selçuk, Turkey | xt20 | KX168785 |  | KX168887 | KX168998 |
| 159 | *Montivipera xanthina* | Selçuk, Turkey | xt21 | KX168786 |  | KX168888 | KX168999 |
| 160 | *Montivipera xanthina* | Selçuk, Turkey | xt22 | KX168787 |  | KX168889 | KX169000 |
| 161 | *Montivipera xanthina* | Selçuk, Turkey | xt23 | KX168788 |  | KX168890 | KX169001 |
| 162 | *Montivipera xanthina* | Selçuk, Turkey | xt24 | KX168789 |  | KX168891 | KX169002 |
| 163 | *Montivipera xanthina* | Olympos, Turkey | xt25 | KX168790 |  | KX168892 | KX169003 |
| 164 | *Montivipera xanthina* | Tekirova, Turkey | xt26 | KX168791 |  | KX168893 | KX169004 |
| 165 | *Montivipera xanthina* | Tekirova, Turkey | xt27 | KX168792 |  | KX168894 | KX169005 |
| 166 | *Montivipera xanthina* | Loutrós, Turkey | xt28 | KX168793 |  | KX168895 | KX169006 |
| 167 | *Montivipera xanthina* | Loutrós, Turkey | xt29 | KX168794 |  | KX168896 | KX169007 |
| 168 | *Montivipera xanthina* | Kos, Turkey | xt30 | KX168795 |  | KX168897 | KX169008 |
| 169 | *Montivipera xanthina* | Kohu Dağ, Turkey | xt31 | KX168796 |  | KX168898 | KX169009 |
| 170 | *Montivipera xanthina* | Kohu Dağ, Turkey | xt32 | KX168797 |  | KX168899 | KX169010 |
| 171 | *Montivipera xanthina* | Kumluca, Turkey | xt36 | KX168798 |  | KX168900 | KX169011 |
| 172 | *Montivipera xanthina* | Tekirova, Turkey | xt37 | KX168799 |  | KX168901 | KX169012 |
| 173 | *Montivipera xanthina* | Kratigos, Lesvos, Turkey | xt40 | KX168801 |  | KX168903 | KX169014 |
| 174 | *Montivipera xanthina* | Ephesos, Turkey | xt42 | KX168802 |  | KX168904 | KX169015 |
| 175 | *Montivipera xanthina* | Loutrós, Turkey | xt45 | KX168803 |  | KX168905 | KX169016 |
| 176 | *Montivipera xanthina* | Loutrós, Turkey | xt46 | KX168906 |  | KX169017 | KX169017 |
| 177 | *Montivipera xanthina* | Ephesos, Turkey | x52 | KX168808 |  | KX168910 | KX169021 |
| 178 | *Macrovipera lebetina* |  | lb14 | KJ415300 |  |  |  |
| 179 | *Macrovipera razii* |  | ERP_1981 | MF445994 |  |  |  |


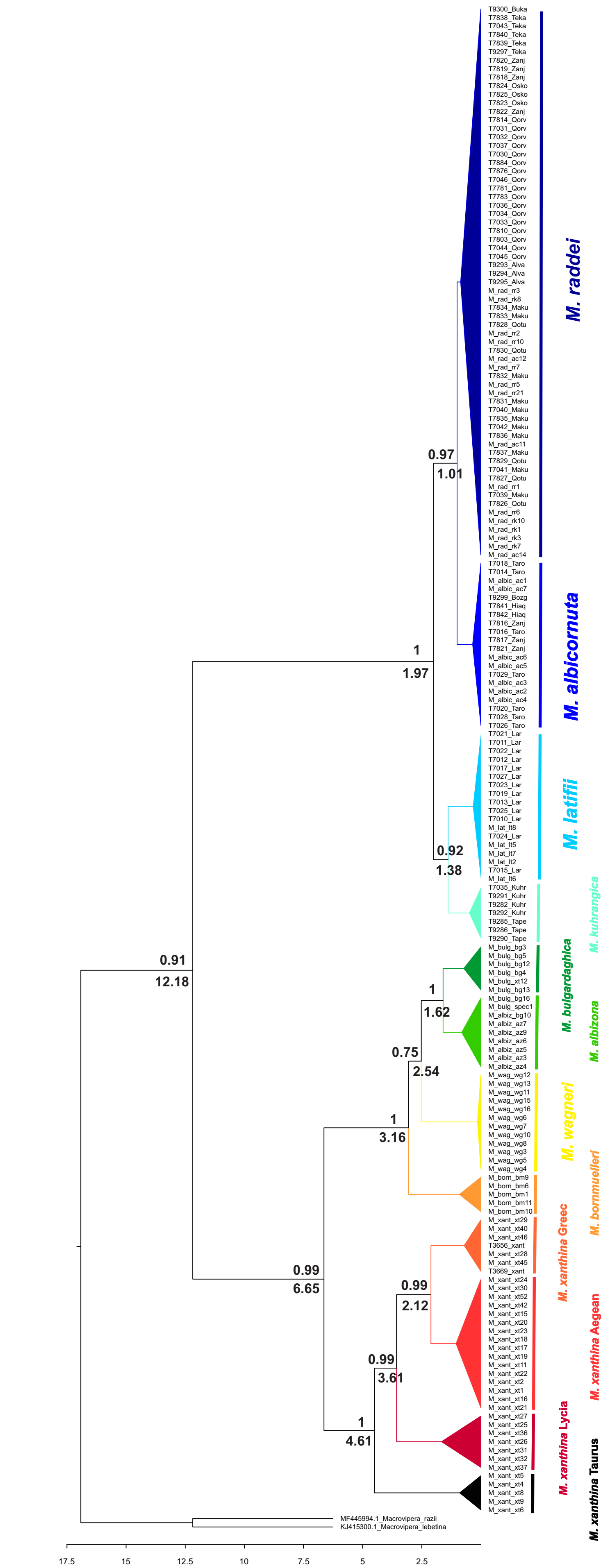


**Fig. S1.** The Bayesian phylogeny tree reconstructed from the 177 sequences of mountain vipers (*Montivipera*). The tree was reconstructed based on a partitioned analysis with three Mitochondrial genes (CYTB, COX1, ND5). Values above and below the branches are the posterior probabilities and mean age estimates, respectively. Colors of the branches corresponds to the geographic origin of the species as shown in Fig. 1.
